# Molecular characteristics and pathogenicity of a novel chicken astrovirus variant

**DOI:** 10.1186/s13567-023-01250-1

**Published:** 2023-12-08

**Authors:** Xiaoqing Bi, Zhenrui Song, Fanrun Meng, Shiwei Sun, Xusheng Du, Mengzan Yang, Defang Zhou, Xiangyu Cheng, Longying Ding, Hengyang Shi, Feng Lang, Huaibiao Luan, Bing Deng, Liangyu Yang, Ziqiang Cheng

**Affiliations:** 1 College of Veterinary Medicine, Shandong Agricultural University, Tai’an, 271018 China; 2 College of Agricultural Science and Engineering, Liaocheng University, Liaocheng, 252000 China; 3 Qingdao Yibang Bioengineering Co, Qingdao, 266000 China; 4 Agricultural and Animal Husbandry Science Research and Promotion Center of Shigatse City, Shigatse, 857000 China; 5 College of Veterinary Medicine, Yunnan Agricultural University, Kunming, 650000 China

**Keywords:** Chicken astrovirus, molecular characterization, sequence analysis, pathogenic analysis

## Abstract

It is well-established that the genetic diversity, regional prevalence, and broad host range of astroviruses significantly impact the poultry industry. In July 2022, a small-scale commercial broiler farm in China reported cases of growth retardation and a 3% mortality rate. From chickens displaying proventriculitis and pancreatitis, three chicken astroviruses (CAstV) isolates were obtained and named SDAU2022-1-3. Complete genomic sequencing and analysis revealed the unique characteristics of these isolates from known CAstV strains in ORF1a, ORF1b, and ORF2 genes, characterized by an unusually high variability. Analysis of amino acid mutations in ORF1a, ORF1b, and ORF2 indicated that the accumulation of these mutations played a pivotal role in the emergence of the variant strain. Inoculation experiments demonstrated that affected chickens exhibited liver and kidney enlargement, localized proventricular hemorrhage, and a dark reddish-brown appearance in about two-thirds of the pancreas. Histopathological examination unveiled hepatic lymphocytic infiltration, renal tubular epithelial cell swelling, along with lymphocytic proventriculitis and pancreatitis. Quantitative reverse transcription-polymerase chain reaction (qRT-PCR) analysis indicated viremia and viral shedding at 3 days post-infection (dpi). The proventriculus displayed the highest viral loads, followed by the liver, kidney, duodenum, and pancreas. Liver parameters (AST and ALT) and kidney parameters (UA and UN) demonstrated mild damage consistent with earlier findings. While the possibility of new mutations in the ORF2 gene of CAstV causing proventriculitis and pancreatitis warrants further investigation, these findings deepen our comprehension of CAstV’s pathogenicity in chickens. Additionally, they serve as valuable references for subsequent research endeavors.

**Supplementary Information:**

The online version contains supplementary material available at 10.1186/s13567-023-01250-1.

## Introduction

Astroviruses are non-enveloped, single-stranded, positive-sense RNA viruses with diameters ranging from approximately 28–30 nm and genome lengths of 6.11–7.72 kb (excluding poly(A) tails) [1]. These viruses share a common genome structure featuring three open reading frames (ORFs): ORFla, ORF1b, and ORF2, accompanied by 5′ and 3′ untranslated regions (UTRs) [2]. The overlapping region between ORF1a and ORF1b, marked by a conserved ribosomal shift frame signal sequence (5′- AAAAAAC-3′) and an adjacent hairpin structure, encodes non-structural proteins [3]. The translation of the downstream RNA-dependent RNA polymerase (RdRp) relies on the ribosome shift signal sequence [4] and also governs the synthesis of the ORF1ab fusion protein [5]. It is now understood that the ORF2 gene, responsible for the viral capsid protein, displays the highest variability, influencing antigenicity and pathogenicity. The protein's N-terminal part is well-conserved, while the C-terminal segment exhibits pronounced variation. The N-terminal portion forms the shell of the coat protein, encapsulating the RNA, while the C-terminal region includes the fibronectin protein [6, 7]. The 3'UTR region contains a conserved “s2m”-like stem-loop structure crucial for viral replication[8].

Avian astrovirus (AAstV), a member of the *Astroviridae* family with various subtypes, plays a significant role in poultry farming. It affects poultry performance and triggers conditions like nephritis in chickens, viral hepatitis in ducks, developmental delay syndrome, and visceral gout [9–12]. It occasionally leads to encephalitis and can infect humans [13–15]. Astrovirus infections in poultry contribute to substantial morbidity and mortality, impaired feed conversion rates, and growth inhibition [16]. Their wide genetic diversity and recombination capacity contribute to the ability to cause extensive disease in diverse hosts [17, 18], leading to considerable economic losses in the poultry industry. Astroviruses exhibit high cross-species transmission in poultry [19], affecting most avian species, including chickens, ducks, geese, and turkeys [20–23]. In chickens, two distinct strains of astrovirus have been identified: avian nephritis virus (ANV) [24] and chicken astrovirus (CAstV) [25]. CAstV infections have been documented globally in countries such as China, the Netherlands, the United States, and Canada [26–28]. Additionally, co-infections with other enteroviruses exacerbate economic losses. CAstV can be vertically transmitted from parents to offspring, reducing chicken embryo hatchability. Horizontal transmission occurs primarily through fecal–oral routes. No vaccines or specific treatments are available to prevent or manage CAstV infection.

In recent years, variant strains of CAstV have emerged worldwide, significantly impacting chicken production performance. Here, we successfully isolated a CAstV strain from commercially raised broilers displaying growth retardation. By analyzing this variant strain’s evolutionary relationships and amino acid mutation sites compared to other known variants, we unveil insights into its potential origin and evolutionary drivers. Furthermore, we investigated the pathogenicity of this strain in specific pathogen-free (SPF) chickens, exploring two infection routes, thus providing a scientific foundation for addressing newly emerging CAstV variants.

## Materials and methods

### Case history

In July 2022, a small-scale independent commercial broiler farm in Shandong Province, China, with approximately 12 000 chickens, reported cases of growth retardation. About 3% of 2-week-old broilers exhibited localized proventricular hemorrhage, abnormal growth, and weight loss. The affected broilers experienced growth retardation starting from 7 days of age. Tissue samples of the proventriculus, duodenum, and pancreas were collected from 10 chickens displaying abnormal growth for laboratory testing. Gross pathological examination revealed local hemorrhage in the proventriculus, with varying degrees observed in the duodenum and pancreas (Additional file 1).

### Cell line and specific pathogen free (SPF) chicken

LMH (Chicken Liver Cancer Cells, iCell-c031) monolayer cells were cultured in DMEM supplemented with 10% FBS and 1% antibiotics (penicillin, streptomycin). The cells were cultured following standard cell culture protocols and maintained at 37 °C in a 5% CO_2_ incubator. One-day-old SPF chickens were obtained from Jinan SPAFAS Poultry Company.

### Virus isolation

Proventriculus samples from ten commercial broilers were homogenized in DMEM using a sonicator. We used an ultrasonic cell disruptor (from Ningbo Xinzhi Biotechnology Co., Ltd.), set at a power of 300w, operating at 4 °C for 5 s with a 10-s interval. Total RNA was extracted from the homogenized samples using TRIzol reagent (Invitrogen, Carlsbad, CA, USA) and then reverse transcribed into cDNA using the TIANScript II RT Kit (TIANGEN, Beijing, China). PCR was used to identify avian influenza virus (AIV), avian orthoreovirus (ARV), chicken anemia virus (CAV), chicken parvovirus (ChPV), infectious bronchitis virus (IBV), avian rotavirus (AvRV), avian nephritis virus (ANV), and chicken astrovirus (CAstV) [29] (Additional file 2C). The tissue homogenates underwent gradient centrifugation at 4 °C, and the resulting supernatant was used to infect LMH monolayer cells. Upon observing marked cytopathic effects (CPE), the cell cultures were harvested through 3 cycles of freeze-thawing. Cell total RNA was extracted 6, 12, 24, 48, and 72 h post-infection (hpi) for CAstV copy number detection using qRT-PCR.

### Quantitative real-time polymerase chain reaction

To assess SDAU2022-1 viral loads at 6, 12, 24, 48, and 72 hpi, a specific primer pair (5′-TCTTCAGCAGCAGCATAC-3′ and 5′-ATGTTGGCGTTCCTAATGT-3') was utilized to amplify a 193 bp fragment of the RdRp gene, which was cloned into the pUC57 vector (Promega, USA) for establishing a qRT-PCR assay standard curve. The standard plasmids were diluted from 1 × 10^8^ to 1 × 10^1^ copies/μL to establish the standard curve. The specificity and sensitivity of the assay were evaluated as per established methods [30]. SYBR Green Master I mix (AG, Hunan, China) was used for qRT-PCR analysis following the manufacturer’s instructions.

### Complete genome amplification and sequencing

To amplify the complete CAstV genome, total RNA was extracted from CAstV-positive cell samples. Extracted RNA was reverse transcribed into cDNA. Six pairs of primers (Additional file 2A) were employed to amplify viral genomic fragments. Amplicons were purified using the PCR clean-up kit (Beyotime, Shanghai, China, Catalog Number: D0033) and ligated into the pGEM-T vector for subsequent sequencing analysis.

### Sequencing analysis

Homology analysis aligned whole-gene and viral structural component sequences using the Clustal Method in MegAlign software. Phylogenetic analysis was conducted using the neighbor-joining method in MEGA 7 with 1000 bootstrap replicates [31]. GenBank accession numbers for the strains used are listed in Additional file 2B.

### Pathogenicity experiment

To investigate CAstV pathogenicity via intraperitoneal and oral infection routes, 90 SPF chickens at 1 day of age were selected. They were divided into intraperitoneal infection, oral infection, and control groups, each containing 30 chickens. Infection and control groups were housed separately in different rooms to ensure isolation. Infected groups were exposed to the CAstV strain through 0.2 mL inoculation at a concentration of 10^–3.506^ TCID_50_/0.2 mL via intraperitoneal and oral routes, respectively. Control chickens received an equivalent PBS dosage. Chickens were managed following 12 national procedures and biosecurity guidelines. Chicken weight was recorded at 3, 5, 7, 10, 15, 20, 25, and 30 dpi, and serum and cloacal swab samples were collected. Three chickens from each group were euthanized for tissue samples (heart, liver, spleen, kidney, thymus, bursa of Fabricius, pancreas, and intestine) to evaluate pathogenicity, viral loads, biochemical parameters (ALT, AST, UA, UN), and histopathological lesions. These four biochemical parameters were measured using biochemical assay kits (Nanjing Jiancheng, China) with serum from different age groups of control and two experimental groups. The OD values were determined the Thermo Scientific Microplate Reader.

### Statistical analysis

Statistical analyses were performed using Prism 9.0 software, employing student *t*-tests or one-way ANOVA to determine differences (GraphPad Software, La Jolla, CA, USA). Results were presented as means ± standard deviations. *P* values < 0.05 were considered different, and *P* values < 0.01 were significantly different. *P* values less than 0.05, 0.01, and 0.001 were denoted as *, **, and ***, respectively.

## Results

### CAstV isolation and identification

**Figure 1:**
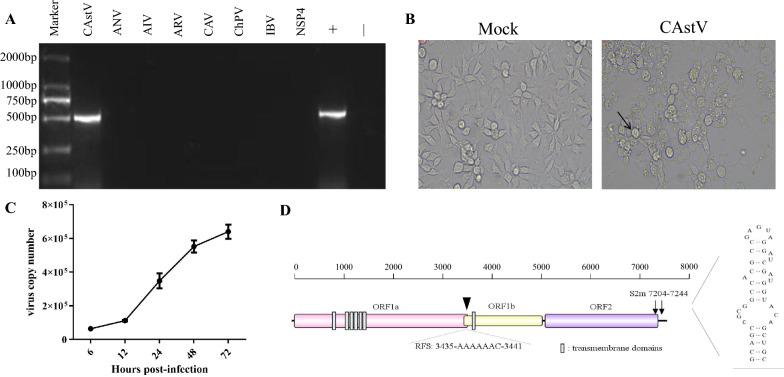
**Isolation and identification of CAstV.** **A** LMHs were infected with virus isolates and tested by PCR for various chicken-original viruses. **B** Cytopathic effects of LMH-infected SDAU2022-1 at 48 hpi. **C** Growth kinetics of CAstV isolate infected LMHs at 6, 12, 24, 48, and 72 hpi. **D** Structural representation of CAstV, featuring three open reading frames (ORFs) and characteristic “stem-loop-II-like” motifs. The initiation site of the heptameric AAAAAAC (RFS) sequence is indicated by black triangles. Gray bars are the transmembrane domains. ORF, open reading frame; RFS, ribosomal frameshift signal; s2m, stem-loop-II-motif.

PCR analysis of proventriculus samples indicated that three chickens exhibited a specific CAstV fragment, while all samples were negative for ANV, AIV, ARV, CAV, ChPV, IBV, and AvRV, confirming CAstV infection (Figure 1A). The filtrate from these positive samples induced a prominent cytopathic effect upon infecting LMH cells, characterized by enlarged, rounded, and clustered cells with a balloon-like appearance during the initial stages of passage 4 (Figure 1B). Additionally, viral loads displayed a characteristic growth curve (Figure 1C).

### Predicted genome organization

The three CAstV isolates, named SDAU2022-1-3, were successfully assembled via overlapping PCR and sequencing. SDAU2022-1 and SDAU2022-2 shared 99.5% nucleotide homology, while SDAU2022-1 and SDAU2022-3 shared 99.7%, confirming they are the same strain. Nucleotide sequences of SDAU2022-1-3 isolates were deposited in NCBI GenBank under accession numbers (OR286520, OR286521, and OR286522). All three isolates had a length of 7331 nt, including a 5′UTR of 21 nt and a 3′UTR of 108 nt, excluding the poly(A) tail.

The complete genome sequences of the three isolates exhibited a gene organization consistent with that of other known CAstVs, featuring a standard arrangement of 5'UTR-ORF1a-ORF1b-ORF2-3'UTR. Notably, the 3'UTR contained a conserved stem-loop-II-like motif (Figure 1D), which plays a crucial role in viral replication [32]. Among all the full-length genomes of CAstVs, SDAU2022 isolates displayed the highest nucleotide sequence similarity. When compared to two recently published CAstV strains from Switzerland, namely CAstV/PB15-HI11/Switzerland/2019 and CAstV/PB7-HI6/Switzerland/2019 [33], the similarity levels were 89.8–89.5%, respectively. The amino acid sequences of ORF1a and ORF1b of SDAU2022 isolates shared identities of 85.7–96.9% with the published sequences of other CAstVs. However, the amino acid sequence of ORF2 among these CAstV isolates exhibited high diversity, with similarities ranging from 38.8% to 96.7% (Additional file 2).

### Phylogenetic analyses of SDAU2022 isolates

**Figure 2:**
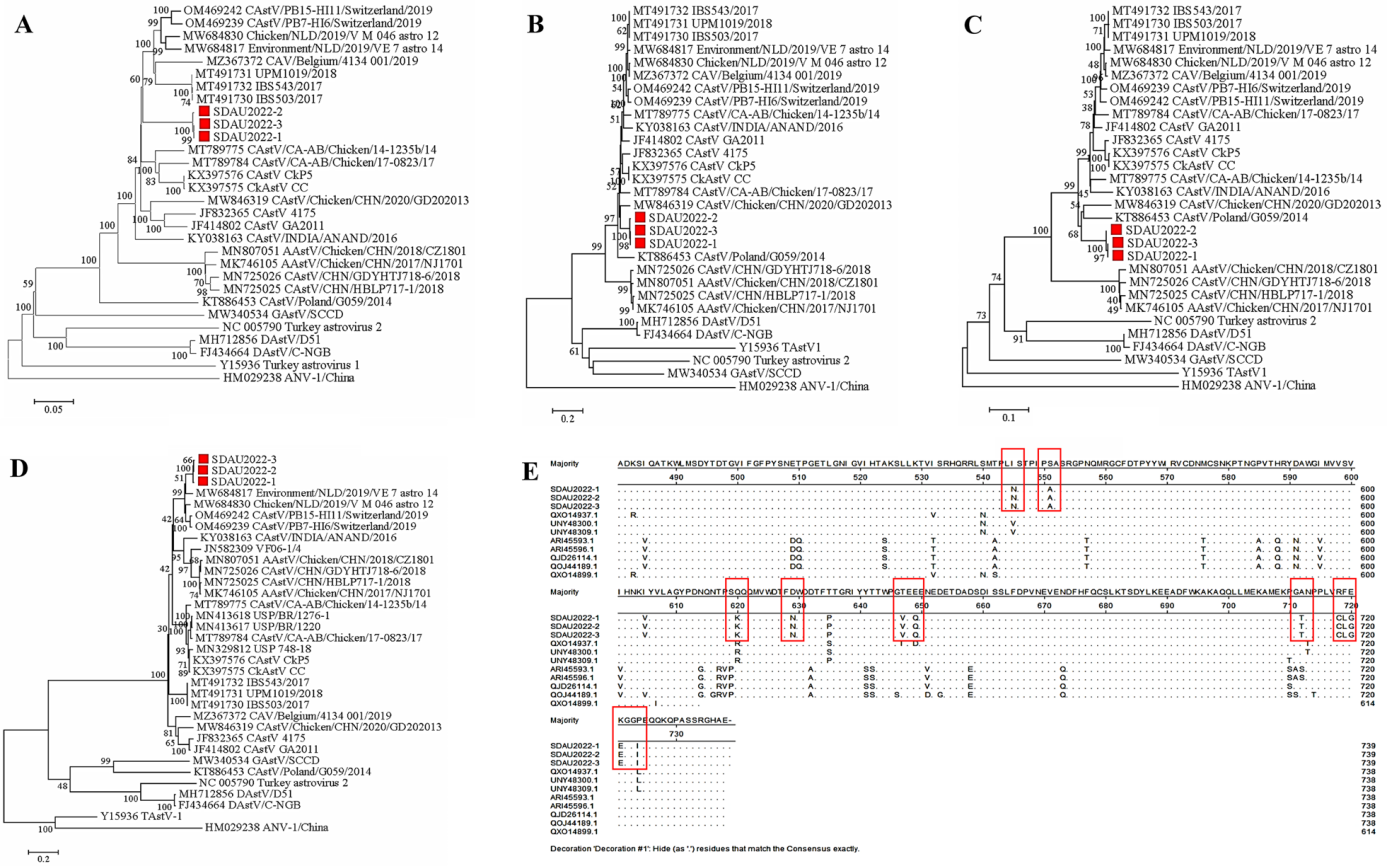
**Phylogenetic relationships between the AAstV isolates**. The analysis was based on the nucleotide sequences of the complete genome (**A**) and amino acid sequences of the complete ORF1a (**B**), ORF1b (**C**), and ORF2 (**D**) regions. The phylogenetic trees were constructed using MEGA 7, the neighbor-joining method, and 1000 bootstrap replicates. GenBank accession numbers of the sequences are indicated in parentheses. The scale bar corresponds to genetic distance. The red squares represent the other three CAstV isolates. **E** The mutation of the amino acid sequences in ORF2 protein.

Phylogenetic analysis was conducted to elucidate the evolutionary relationships between the SDAU2022 isolates and reference strains of CAstV. The phylogenetic trees (Figure 2) were constructed using nucleotide sequences of the whole genome, ORF1a, ORF1b, and ORF2 genes, employing the maximum likelihood method with bootstrapping. The evolutionary branching strongly suggested that the SDAU2022 isolate likely originated from the IBS503/2017 strain in Malaysia. It shared a close relationship with two Canadian strains (CAstV/CA-AB/Chicken/17-823/17 and CAstV/CA-AB/Chicken/14-1235b/14), which trace back to the CkP5 and CC strains isolated from slow-growing chickens in the USA (Figure 2A). In the ORF1a gene, the SDAU2022 isolates clustered with the CAstV/Chicken/CHN/2020/GD202013 strain. Conversely, the amino acid phylogenetic analysis of ORF1b revealed a tight association between the SDAU2022 isolates and the CAstV/Chicken/CHN/2020/GD202013 and CAstV/Poland/G059/2014 strains, forming a sister clade (Figures 2B and C). The ORF2 genes of the SDAU isolates grouped with Environment/NLD/2019/VE_7_astro_14, indicating that the ORF2 gene follows an independent evolutionary pattern (Figure 2D). We also compared the amino acid sequences of ORF1a, ORF1b, and ORF2 with the eight most closely related strains of chicken astroviruses from the database. which revealed 33 amino acid mutations in ORF1a, 17 amino acid mutations in ORF1b, and 22 amino acid mutations in ORF2 (Additional file 3). Moreover, we observed 12 new mutation sites in the ORF2 region. For brevity, we present only the amino acid comparison results of the most mutable region in ORF2 (Figure 2E).

### Clinical symptoms and gross lesions of experimental chickens

**Figure 3:**
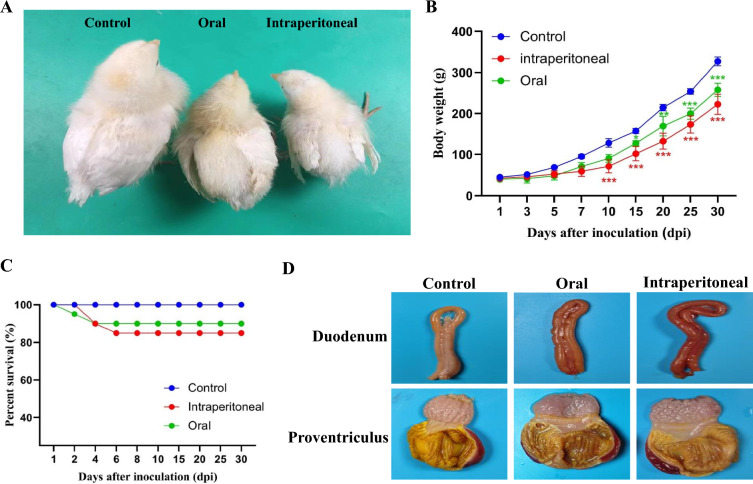
**Body weight gain and survival curves of experimental chickens**. **A** Changes in body weight at 10 days of age in the intraperitoneal and oral groups. Control stands for control. Po stands for oral infection group. Intraperitoneal stands for intraperitoneal infection group. **B** Weight gain of chickens infected with SDAU2022-1. *p < 0.05, ** p < 0.01, *** p < 0.001 **C** Survival curves of chickens after infection. **D** Gross lesions of experimental chickens.

The chickens in the intraperitoneal group displayed more severe loss of appetite and depression than the oral-infected chickens. Additionally, the intraperitoneal infection group exhibited these symptoms earlier than the oral infection group. The chickens infected with the SDAU2022-1 virus experienced significant weight gain inhibition compared to the control group. By the 10^th^ day post-infection, the average weight of the intraperitoneal infection group chickens (71 g) was approximately half that of the control group (128.6 g), while the average weight of the oral infection group chickens was 90.3 g (Figures 3A and B). Mortality rates were 10% in the oral infection group and 15% in the intraperitoneal infection group (Figure 3C). Hemorrhage in the duodenum and pancreas, along with the disappearance of proventriculus papillae and scattered hemorrhagic spots around the proventriculus, were observed in the infected group (Figure 3D). No clinical symptoms or gross lesions were detected in the control group of chickens.

### SDAU2022-1-induced histopathological changes

**Figure 4:**
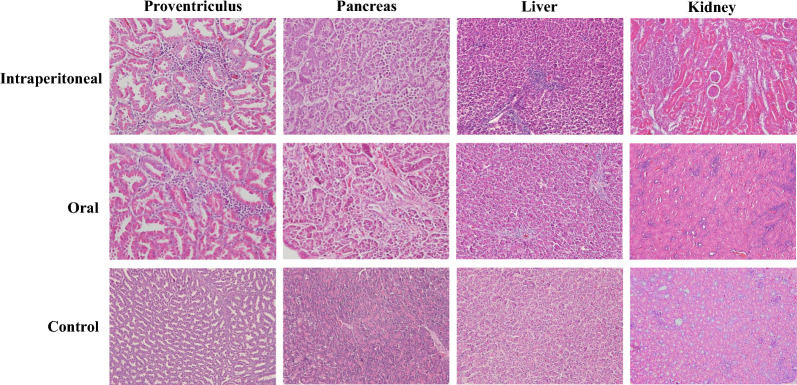
**Histopathologic observations of proventriculus, pancreas, kidney, and liver (H&E staining).** In the intraperitoneal infection group, proventriculus tissue exhibited necrosis of proventriculus tubules accompanied by lymphocytic infiltration (400 ×). Increased density of lymphocytic infiltration in pancreatic tissue (400 ×), while liver tissue displayed lymphocytic infiltration around the central hepatic venous vessels. Kidney tissue presented with necrosis of renal tubular epithelial cells and necrotic foci (200 ×). In the oral infection group, proventriculus tubules exhibited lymphocytic infiltration and proventricular dilatation (400 ×). Pancreatic tissue showed lymphocytic infiltration accompanied by pancreatic hemorrhage (400 ×). Liver tissue displayed lymphocytic infiltration around the central hepatic venous vessels. Kidney tissue exhibited swelling of renal tubular epithelial cells that compressed the lumen of the renal tubules (200 ×). Normal tissue (proventriculus, pancreas, liver, and kidney) in the control group (200 ×).

Chickens in the intraperitoneal and oral infection groups exhibited similar pathological changes following inoculation. Histopathological examination revealed that the most severe lesions caused by this strain were observed in the proventriculus, liver, kidney, pancreas, duodenum, and spleen. In both experimental groups, the proventriculus exhibited epithelial cell exfoliation and lymphocyte infiltration, with the intraperitoneal infection group’s chickens also showing proventricular dilatation. The pancreas of chickens in both groups exhibited hemorrhage and necrosis of glandular cells, accompanied by lymphocyte infiltration. In both experimental groups, lymphocyte infiltration around blood vessels in the liver was evident. In the intraperitoneal infection group, the kidneys of chickens displayed detachment of renal tubular epithelial cells with necrotic foci, while in the oral infection group, the kidneys were predominantly characterized by swelling (Figure 4). Furthermore, both experimental groups exhibited lesions characterized by villous dissolution and necrosis of intestinal mucosal epithelial cells in the duodenum, with more severe spleen hemorrhage observed in the intraperitoneal infection group (Additional file 4).

### Viral loads in tissues

**Figure 5:**
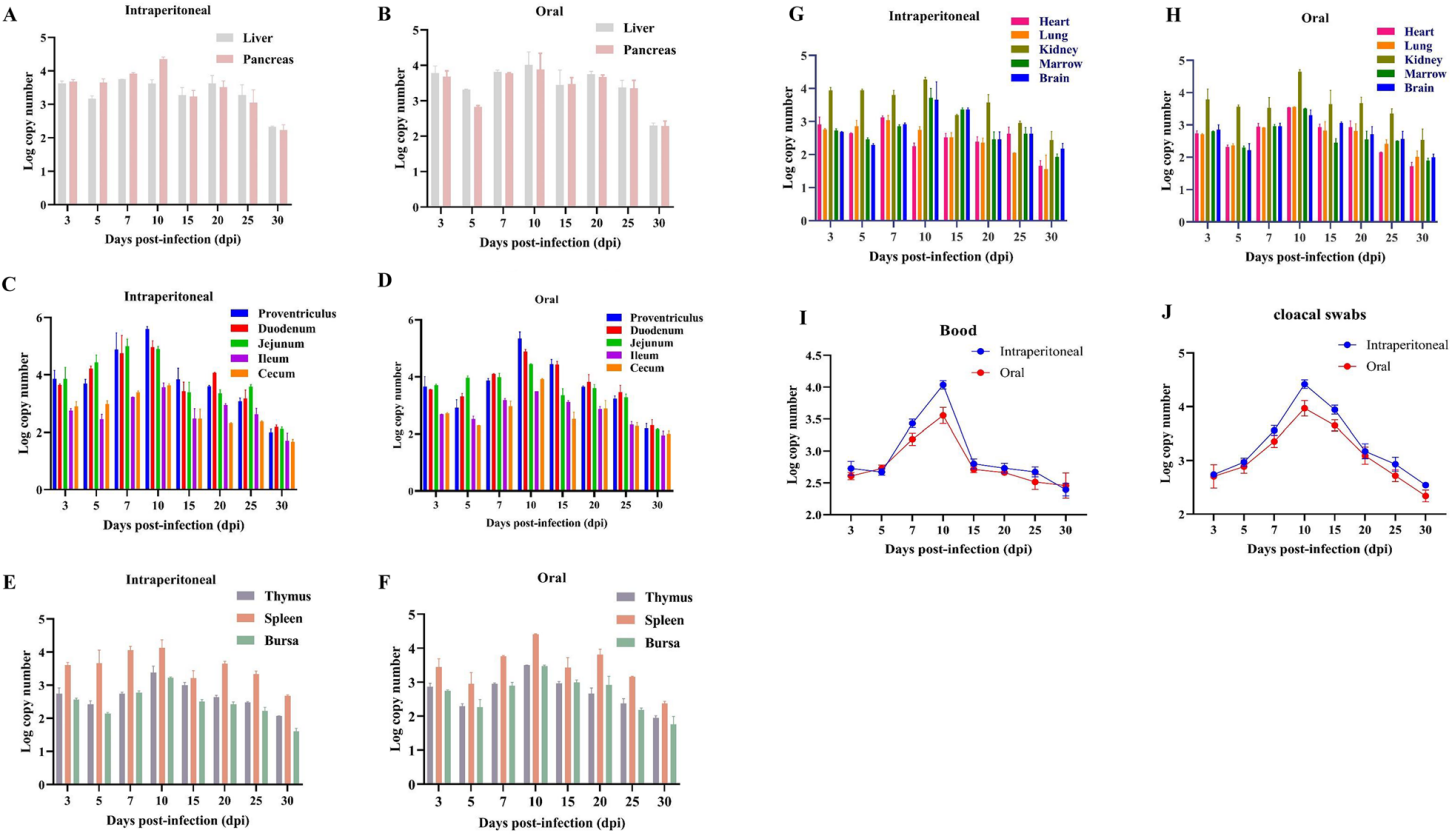
**Detection of viral loads in organs**. Intraperitoneal infection group (**A**) and oral infection group (**B**) of viral loads detection in the digestive organs. Digestive tract viral loads testing in the intraperitoneal group (**C**) and oral group (**D**). Intraperitoneal infection group (**E**) and oral group (**F**) of viral loads detection in the Immune organ. Intraperitoneal infection group (**G**) and oral group (**H**) of viral loads detection in the other organ. **I** The viral loads in blood of chickens infected with CAstV. **J** The viral loads in cloacal swabs of chickens infected with CAstV.

Quantitative real-time polymerase chain reaction (qRT-PCR) was employed to detect the changes in CAstV viral loads in different tissues of chickens. Consistent viral load patterns were observed across all analyzed tissues in both experimental groups, significantly increasing at 3 dpi and 10 dpi in all tissues. The proventriculus displayed the highest viral load, followed by the kidney, duodenum, pancreas, and liver, with copy numbers of approximately 10^4^ (Figures 5A–H). Three days post-inoculation, a rapid increase in viral genomic RNA levels was observed within the digestive system, peaking after 10 days (as shown in Figures 5C, D). This phenomenon was closely associated with the emergence of digestive tract lesions following CAstV infection. Furthermore, viral shedding from the cloaca was observed, and changes in viral genomic RNA levels were detected in the blood of infected chickens at 3 dpi, confirmed by assessing viral genomic RNA in the blood and cloaca at multiple time points (3, 5, 7, 10, 15, 20, 25, and 30 dpi) (Figures 5I, J).

### Liver and kidney damage

**Figure 6:**
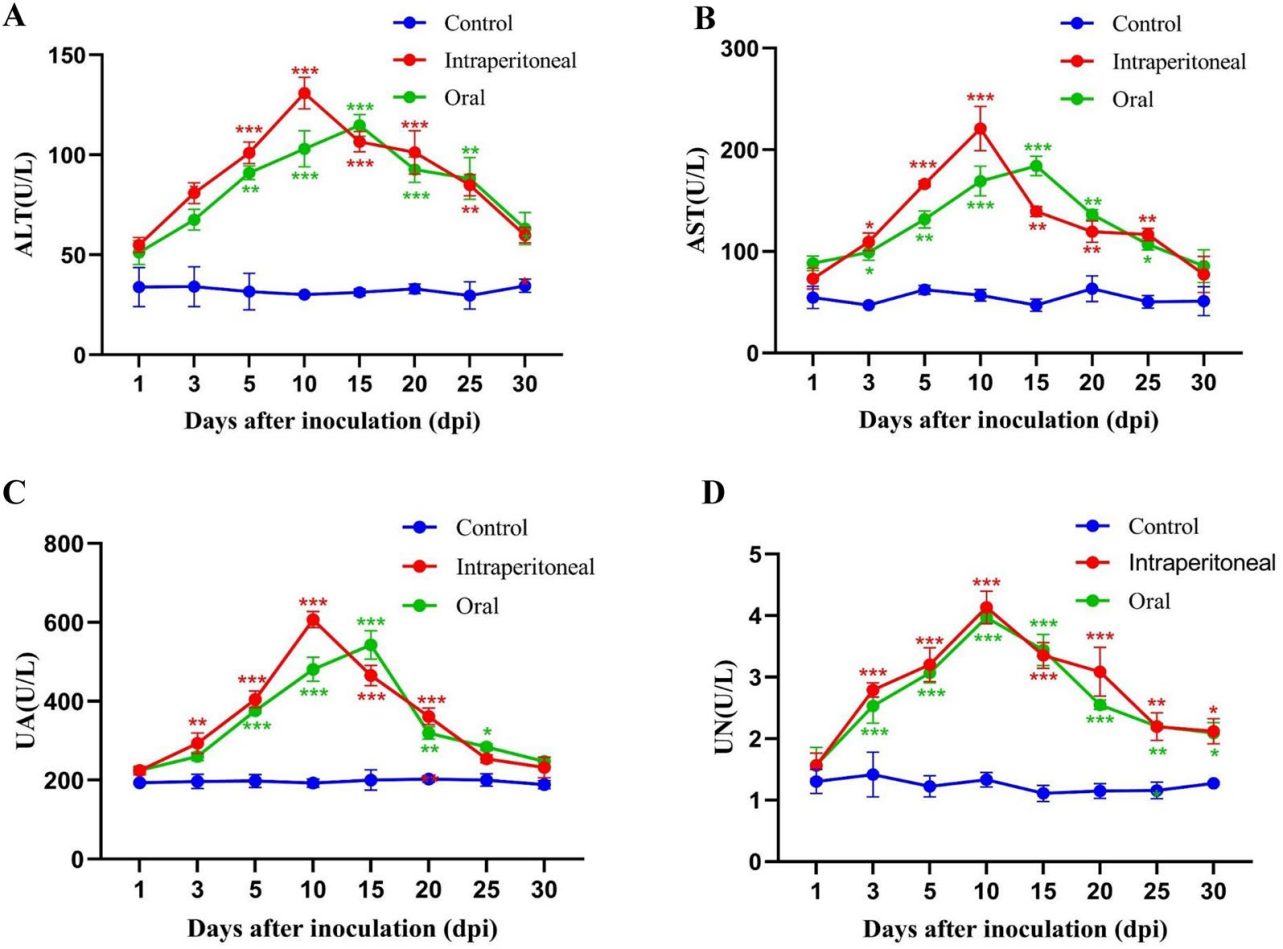
**Serum dynamics of ALT, AST, UA, and UN in chickens post-infection with CAstV.** * Indicates a statistically significant difference between the infection and control groups. ** Indicates a highly significant difference between the infection and control groups. *** Indicates an exceptionally significant difference between the infection and control groups.

The detection results of the three groups are illustrated in Figure 6. The analysis revealed that the levels of alanine aminotransferase (ALT), aspartate transaminase (AST), uric acid (UA), and urea nitrogen (UN) were higher in the infected groups compared to the control group. In the intraperitoneal infection group, ALT, AST, UA, and UN levels peaked at 10 dpi. Similarly, in the oral infection group, ALT, AST, and UA levels peaked at 15 dpi, while UN peaked earlier at 10 dpi. Subsequently, the aforementioned biochemical parameters gradually decreased over time.

## Discussion

Over the years, astroviruses have been identified in over 80 host species, raising concerns about interspecies and even zoonotic transmission [34, 35]. The prevalence of chicken astrovirus now surpasses 45–60% in domestic poultry populations despite limited suitable animal models and unclear pathogenesis [36]. Efficient identification and detection of pathogenic microorganisms are crucial across various regions. In response to the recurring growth retardation issues in commercial broiler farms in Shandong Province, China, which significantly impacted the economic growth of these farms, we conducted virus isolation, identification, and genetic characterization analysis. As a result, we successfully isolated three CAstV strains with a homology of around 99%. Moreover, we employed two inoculation methods to replicate the infection in animals, of which oral infection effectively mimicked the fecal–oral transmission route of chicken astrovirus.

The complete genomes of these CAstV isolates were assembled and analyzed using fragment primers. Like other astroviruses, the viral genome consists of UTR regions at both ends and three ORF proteins in the middle [37]. Previous studies have indicated that viruses carrying the s2m motif spread more rapidly than those lacking it [38]. In this study, we characterized the RNA tertiary structure map of the s2m motif in this strain, which aligned with previously characterized structures [39].

Phylogenetic analysis based on the whole-genome system suggested that the SDAU2022 isolates likely originated from the IBS503/2017 strain in Malaysia. These isolates share a branch with two Canadian strains (CAstV/CA-AB/Chicken/17-0823/17 and CAstV/CA-AB/Chicken/14-1235b/14), which themselves trace back to the CkP5 and CC strains isolated from slow-growing chickens in the USA. This finding suggests that the SDAU2022 isolates might represent an evolutionary variant of the CkP5 or CC strains [40]. However, further research is required to investigate how this strain was introduced to Shandong province. Interestingly, despite substantial nucleotide differences (11.0–20.2%) between the isolated CAstV and the reference strains (10.2–12.9%), the amino acid differences between the genes encoding ORF1a, ORF1b, and ORF2 and the reference strain were small and relatively conserved, with 4.6%, 5.2%, and 4.3% amino acid differences for these three genes, respectively. Base-silencing substitutions considerably minimized differences in amino acid sequences between the isolates and the reference strains. Research has demonstrated that mutations are the primary drivers of RNA viral molecule evolution [41]. In this experiment, we analyzed amino acid mutations in ORF1a, ORF1b, and ORF2 separately, revealing 33, 17, and 22 amino acid mutations, respectively. Additionally, we observed 12 new mutation sites in the ORF2 region. The accumulation of amino acid mutations suggests the potential for astrovirus evolution, leading to the emergence of variant CAstV strains.

Our animal experiments utilizing two inoculation routes yielded the following results. Intraperitoneal infection with SDAU2022-1 induced more pronounced gastrointestinal malabsorption and growth retardation symptoms than oral infection with SDAU2022-1. Moreover, SDAU2022-1 infection led to an increased standard deviation of body weight, implying fluctuating body weight within the group of chickens. Although the mortality of chickens infected with SDAU2022-1 was low, the diminished performance they caused poses a potential risk to the poultry industry. Some researchers have suggested that SDAU2022-1 infection can result in severe visceral gout, interstitial nephritis, and structural disorganization and necrosis of small intestinal epithelial cells [42]. In the present study, the SDAU2022-1 isolate induced relatively mild liver and kidney lesions, mainly characterized by lymphocytic infiltration around hepatic veins, necrosis of renal tubular epithelial cells, and tubular dilatation, with no urate deposition. In recent years, studies have reported CAstV-induced intestinal histological changes, including structural disturbances and necrosis of small intestinal epithelial cells [43]. Our findings extended these observations by uncovering lesions in the duodenum and the proventriculus, marked by necrosis of proventriculus tubules and lymphocyte infiltration. Additionally, we provided hitherto undocumented evidence that the SDAU2022-1 isolate could induce lymphocytic infiltration and necrotic disintegration of acinar cells in the pancreas, possibly due to pancreatic post-secretory outflow obstruction after duodenal injury, leading to pancreatitis. Furthermore, we observed different microscopic damage under the two inoculation routes, with milder lesions in the orally infected group due to gastrointestinal first-pass and hepatic first-pass metabolism. These results suggest that the SDAU2022-1 isolate primarily causes proventriculitis and pancreatitis, disrupting chicken metabolism and resulting in delayed growth, reduced evenness, and decreased feed conversion rate.

According to the tissue viral load analysis results, viral loads were detected in the blood, cloacal swabs, and various organs of infected chicks at 3 dpi, which suggests that viremia could develop within 72 h post-inoculation [44]. Studies have indicated that the main site of CAstV replication is the proventriculus, with other potential target organs, including the pancreas, duodenum, kidneys, and liver, which might exhibit higher viral loads. Notably, the proventriculus showed the highest viral loads in both experimental groups, differing from other strain variations [45]. Our study observed that the virus had the highest viral loads in the digestive system, implying that CAstV can infect chicks through the digestive system [46]. Enhancing disinfection measures in the chicken house during peak virus concentration is vital to minimize potential disease transmission. These findings shed light on CAstV transmission routes, aiding our understanding of its spread.

Besides, liver and kidney damage indicators demonstrated that in the intraperitoneal infection group, AST and ALT levels peaked earlier than in the oral infection group, indicating that the extent of hepatocellular damage is influenced by the infection route [47, 48]. UA and UN levels can reflect the degree of renal damage [49], and nearly all blood biochemical parameters were higher in both experimental groups than in the control group, decreasing over time.

In summary, we isolated a variant strain of CAstV and unraveled its possible origin and distinct genetic characteristics. The accumulation of amino acid mutations in ORF2 likely played a pivotal role in the evolution of SDAU2022 isolates. This mutant strain of CAstV can incite proventriculitis and pancreatitis in chickens. However, further investigation is necessary to determine whether these differences can be attributed to new mutations in ORF2. Importantly, our study provides a foundation for clinical protection and control strategies against astrovirus-induced proventriculitis.

### Supplementary Information

**Additional file 1: Clinical manifestation of chickens during autopsy. A All deceased chickens were lighter in weight.** **B** Proventriculus enlargement with localized hemorrhage (histological image from the farm). **C** Duodenal and pancreatic hemorrhage (histological image of the submitted sample).

**Additional file 2: Primer sequences and comparisons of nucleotide and amino acid sequences of CAstV.** **A** Primer sequence for the complete genome. **B** Comparisons of nucleotide and amino acid sequences of CAstV SDAU2022 with selected representative astroviruses. **C** Primers were used in this study for the detection of viruses.

**Additional file 3: Number of amino acid mutations in ORF1a, ORF1b, and ORF2 (amino acid comparisons with the eight nearest chicken astroviruses with homology).**

**Additional file 4: Histological examination of tissue samples (HE, 200×).** The intraperitoneal and oral infection groups exhibited villus dissolution, necrosis of mucosal epithelial cells, and congestive dilatation of the mucosal muscular layer in the duodenal tissues. Splenic hemorrhage in the intraperitoneal infection group and the oral infection group. Normal spleen and duodenal tissue in the control group.

## Data Availability

The nucleotide sequences of CAstV isolates were deposited in the NCBI GenBank under accession numbers (OR286520, OR286521, OR286522).
