# Supplementary material for: Molecular characteristics and pathogenicity of a novel chicken astrovirus variant
Source: Vet Res. 2023 Dec 8;54:117. doi: 10.1186/s13567-023-01250-1 (PMC10709865; doi:10.1186/s13567-023-01250-1)
Supplement: Supplementary file 1 — Additional file 1: Clinical manifestation of chickens during autopsy. A All deceased chickens were lighter in weight. B Proventriculus enlargement with localized hemorrhage (histological image from the farm). C Duodenal and pancreatic hemorrhage (histological image of the submitted sample). [file 13567_2023_1250_MOESM1_ESM.docx]

**Additional file 1 Clinical manifestation of chickens during autopsy.**


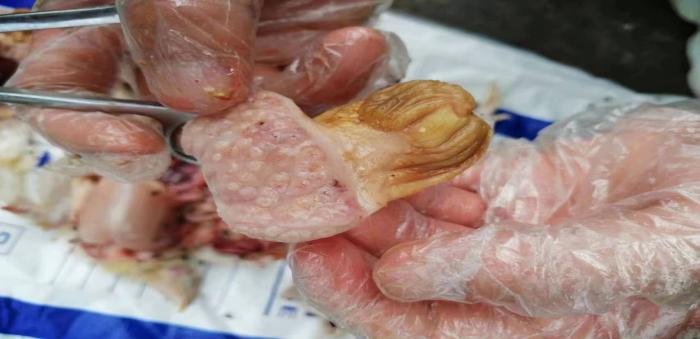

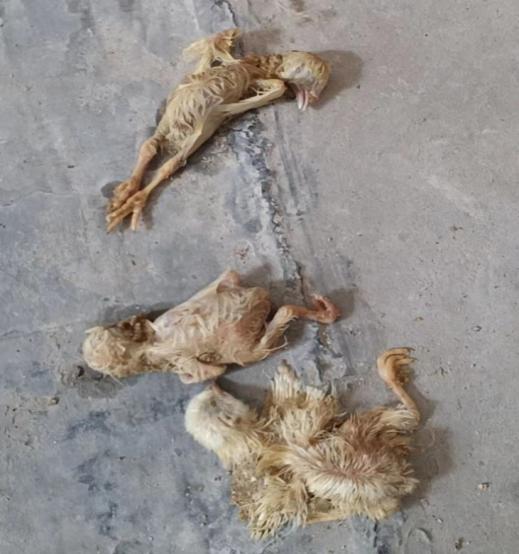


**A**

**B**


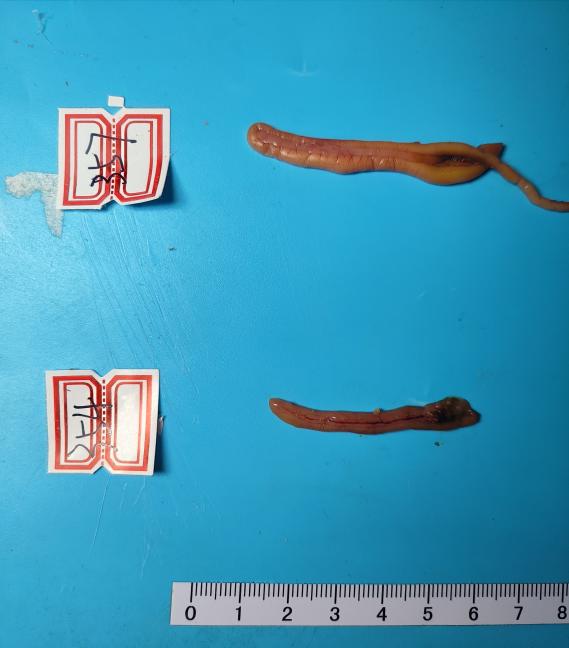


**C**

(A) All deceased chickens were lighter in weight. (B) Proventriculus enlargement with localized hemorrhage (histological image from the farm). (C) Duodenal and pancreatic hemorrhage (histological image of the submitted sample).
