# Supplementary material for: Molecular characteristics and pathogenicity of a novel chicken astrovirus variant
Source: Vet Res. 2023 Dec 8;54:117. doi: 10.1186/s13567-023-01250-1 (PMC10709865; doi:10.1186/s13567-023-01250-1)
Supplement: Supplementary file 2 — Additional file 2: Primer sequences and comparisons of nucleotide and amino acid sequences of CAstV. A Primer sequence for the complete genome. B Comparisons of nucleotide and amino acid sequences of CAstV SDAU2022 with selected representative astroviruses. C Primers were used in this study for the detection of viruses. [file 13567_2023_1250_MOESM2_ESM.docx]

**Additional file 2.**

**Additional file 2A** **Primer sequence for the complete genome.**

| **Primer** | **Sequence** | **Length(bp)** |
| --- | --- | --- |
| 1 | F: GAGGGTGTGGGCGATGGC  R: GCTGTTCACTATTAAAAGCACTACG | 947 bp |
| 2 | F: AAGTGCTACAACACTCATGGGAACG  R: TCCATTCCGCGTGATGGTCTCAA | 2,176 bp |
| 3 | F: GGCAGAGGATTGGTATGAC  R: CAACCACAACAAGCAAGG | 1268bp |
| 4 | F: AGAATCCACACCAGCATAC  R: GTAACTGCCATGCGATCA | 1416bp |
| 5 | F: ATGAGCGTAGAGATAGGAGA  R: ACATAATCATCGCCAGCAT | 930bp |
| 6 | F: CTGAGCAGCAAAAACAACCT  R: AAATGCCAATTAATTTAATTCAAAA | 594bp |

**Additional file 2B Comparisons of nucleotide and amino acid sequences of CAstV SDAU2022 with selected representative astroviruses.**

| Avian astrovirus isolates | Accession Number | Sequence identity (%) | | | | Genetic distance |
| --- | --- | --- | --- | --- | --- | --- |
|  |  | Genome (nt) | ORF1a (aa) | ORF1b (aa) | ORF2 (aa) | ORF2 (aa) |
| CAstV/PB15-HI11/Switzerland/2019 | OM469242.1 | 89.8 | 95.4 | 94.8 | 95.7 | 0.043 |
| CAstV/PB7-HI6/Switzerland/2019 | OM469239.1 | 89.5 | 95.3 | 95.2 | 95.7 | 0.043 |
| Chicken/NLD/2019/V_M_046_astro_12 | MW684830.1 | 89.4 | 95.4 | 95.6 | 95.7 | 0.043 |
| Environment/NLD/2019/VE_7_astro_14 | MW684817.1 | 89.1 | 95.4 | 95.2 | 96.7 | 0.033 |
| CAstV/CA-AB/Chicken/17-0823/17 | MT789784.1 | 87.2 | 95.0 | 95.8 | 89.8 | 0.102 |
| CkP5 | KX397576.1 | 87.1 | 95.8 | 96.3 | 90.4 | 0.096 |
| CC_CkAstV | KX397575.1 | 87.0 | 95.7 | 96.3 | 90.2 | 0.098 |
| CAstV/CA-AB/Chicken/14-1235b/14 | MT789775.1 | 86.7 | 95.0 | 95.8 | 90.0 | 0.100 |
| GA2011 | JF414802.1 | 85.5 | 95.2 | 96.3 | 83.6 | 0.164 |
| CAstV/Chicken/CHN/2020/GD202013 | MW846319.1 | 85.3 | 95.0 | 96.7 | 84.0 | 0.160 |
| UPM1019/2018 | MT491731.1 | 85.3 | 95.8 | 95.4 | 87.7 | 0.123 |
| IBS543/2017 | MT491732.1 | 85.2 | 95.8 | 95.4 | 87.7 | 0.123 |
| 4175 | JF832365.1 | 85.2 | 95.3 | 86.2 | 79.7 | 0.205 |
| IBS503/2017 | MT491730.1 | 85.1 | 95.9 | 95.2 | 87.7 | 0.123 |
| CAstV/INDIA/ANAND/2016 | KY038163.1 | 85.0 | 94.6 | 96.1 | 87.1 | 0.129 |
| CAV/Belgium/4134_001/2019 | MZ367372.1 | 84.1 | 95.5 | 95.4 | 79.1 | 0.113 |
| AAstV/Chicken/CHN/2017/NJ1701 | MK746105.2 | 77.9 | 86.4 | 86.1 | 86.2 | 0.138 |
| AAstV/Chicken/CHN/2018/CZ1801 | MN807051.1 | 77.7 | 86.5 | 86.1 | 86.3 | 0.137 |
| CAstV/CHN/GDYHTJ718-6/2018 | MN725026.1 | 77.6 | 86.5 | 85.7 | 86.2 | 0.138 |
| CAstV/CHN/HBLP717-1/2018 | MN725025.1 | 77.6 | 86.7 | 85.9 | 86.0 | 0.140 |
| CAstV/Poland/G059/2014 | KT886453.1 | 76.1 | 93.8 | 96.9 | 38.8 | 0.615 |
| USP/BR/1220 | MN413617.1 | — | — | — | 89.6 | 0.104 |
| USP 748-18 | MN329812.1 | — | — | — | 89.7 | 0.103 |
| USP/BR/1276-1 | MN413618.1 | — | — | — | 90.0 | 0.100 |
| VF06-1/4 | JN582309.1 | — | — | — | 87.0 | 0.130 |
| TAstV-1 | Y15936.2 | 52.6 | 40.5 | 57.1 | 36.4 | 0.640 |
| TAstV-2 | NC_005790.1 | 54.5 | 46.2 | 69.4 | 36.1 | 0.622 |
| DAstV/C-NGB | FJ434664.1 | 58.1 | 57.7 | 69.8 | 36.0 | 0.623 |
| DAstV/D51 | MH712856.1 | 58.1 | 57.5 | 70.0 | 35.8 | 0.624 |
| GAstV/SCCD | MW340534.1 | 57.8 | 49.7 | 66.1 | 39.1 | 0.612 |
| ANV-1/China | HM029238.1 | 48.7 | 26.6 | 53.4 | 28.1 | 0.722 |

**Additional file 2C Primers were used in this study for the detection of viruses.**

| **Primer** | **Sequence (5’→3’)** | **Length (bp)** |
| --- | --- | --- |
| CAstV | F: KCATGGCTYCACCGYAADCA  R: CGGTCCATCCCTCTACCAGATTT | 510bp |
| ANV | F: GYTGGGCGCYTCYTTTGAYAC  R: CRTTTGCCCKRTARTCTTTRT | 473 bp |
| AIV | F: GCCATCCTAGCAACGACTGT  R: AGGATCATGGACGGATTACGA | 1275bp |
| ARV | F: AGTCGATTAGGTACGATGCCA  R: GTAACTGCCATGCGATCA | 685bp |
| CAV | F: GGCTACTATTCCATCACCATTCT  R: GCTCGTCTTGCCATGTTACA | 831bp |
| ChPV | F: TTCTAATAACGATATCACT  R: TTTGCGCTTGCGGTGAAGTCTGGCTCG | 561bp |
| IBV | F: GCGAAAACTGAACAAAAGAC  R: GGCCATAACTAACATAAGGG | 1700bp |
| NSP4 | F: GTGCGGAAAGATGGAGAAC  R: GTTGGGGTACCAGGGATTAA | 630bp |
