# Supplementary material for: Molecular characteristics and pathogenicity of a novel chicken astrovirus variant
Source: Vet Res. 2023 Dec 8;54:117. doi: 10.1186/s13567-023-01250-1 (PMC10709865; doi:10.1186/s13567-023-01250-1)
Supplement: Supplementary file 3 — Additional file 3: Number of amino acid mutations in ORF1a, ORF1b, and ORF2 (amino acid comparisons with the eight nearest chicken astroviruses with homology). [file 13567_2023_1250_MOESM3_ESM.docx]

**Additional file 3 Number of amino acid mutations in ORF1a, ORF1b, and ORF2 (amino acid comparisons with the eight nearest chicken astroviruses with homology).**

| genes | Amino acid mutation sites |
| --- | --- |
| ORF1a | 33 |
| ORF1b | 17 |
| ORF2 | 22 |
