# Supplementary material for: Molecular characteristics and pathogenicity of a novel chicken astrovirus variant
Source: Vet Res. 2023 Dec 8;54:117. doi: 10.1186/s13567-023-01250-1 (PMC10709865; doi:10.1186/s13567-023-01250-1)
Supplement: Supplementary file 4 — Additional file 4: Histological examination of tissue samples (HE, 200×). The intraperitoneal and oral infection groups exhibited villus dissolution, necrosis of mucosal epithelial cells, and congestive dilatation of the mucosal muscular layer in the duodenal tissues. Splenic hemorrhage in the intraperitoneal infection group and the oral infection group. Normal spleen and duodenal tissue in the control group. [file 13567_2023_1250_MOESM4_ESM.docx]

**Additional file 4.**

**Duodenum**


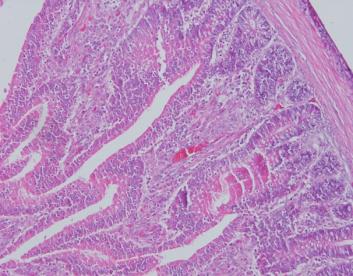

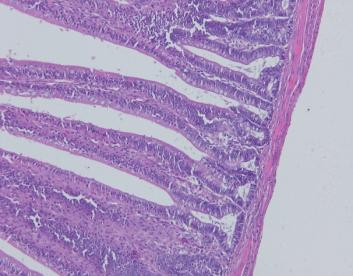

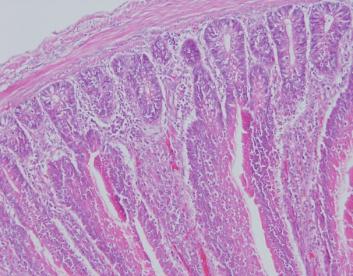


**Spleen**


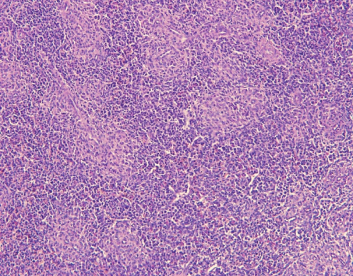

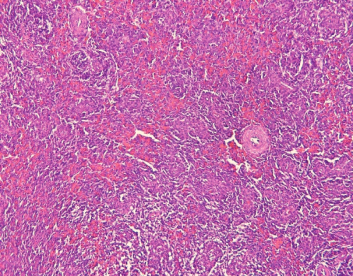

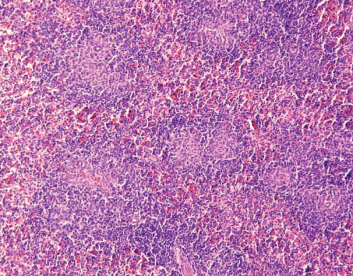


**Intraperitoneal**

**Oral**

**Control**

Histological examination of tissue samples (HE, 200×).

The intraperitoneal and oral infection groups exhibited villus dissolution, necrosis of mucosal epithelial cells, and congestive dilatation of the mucosal muscular layer in the duodenal tissues. Splenic hemorrhage in the intraperitoneal infection group and the oral infection group

Normal spleen and duodenal tissue in the control group.
